# Prefrontal Neurovascular Coupling Abnormalities Associated With Cognitive Performance in Multiple Sclerosis

**DOI:** 10.1002/cns.71124

**Published:** 2026-08-31

**Authors:** Ruisi Gong, Hao Zhang, Jinlin Jiao, Lei Liu, Xiaohan Xu, Huiwen Song, Yuexin Zhou, Yuanjie Duan, Hefei Fu, Jibin Cao, Lingling Cui

**Affiliations:** ^1^ Department of Radiology The First Hospital of China Medical University Shenyang Liaoning China; ^2^ Department of Neurology The First Hospital of China Medical University Shenyang Liaoning China

**Keywords:** cognitive performance, multimodal MRI, multiple sclerosis, neurovascular coupling, prefrontal cortex

## Abstract

**Background:**

Cognitive impairment is a prevalent and debilitating feature in multiple sclerosis (MS), yet its pathophysiology remains incompletely understood. Dysfunction of the neurovascular unit (NVU) and impaired neurovascular coupling (NVC) may be related to cognitive performance in MS, but this pathway remains insufficiently studied. This study investigated whether NVC abnormalities are associated with cognitive performance in MS.

**Methods:**

Ninety‐seven MS patients and 83 healthy controls (HCs) underwent resting‐state functional magnetic resonance imaging (rs‐fMRI) and arterial spin labeling (ASL) perfusion MRI acquisition. The amplitude of low‐frequency fluctuations (ALFF) was used as an index of regional spontaneous neuronal activity, and cerebral blood flow (CBF) was quantified. NVC was quantified globally (CBF‐ALFF correlation) and regionally (CBF/ALFF ratio). We analyzed the correlations between regional NVC metrics and cognitive performance and Expanded Disability Status Scale (EDSS) scores. Sensitivity analyses tested robustness across alternative functional metrics, gray matter volume (GMV), T2 lesion volume, disability severity, treatment status, and the relapsing‐remitting multiple sclerosis (RRMS) subgroup.

**Results:**

Compared with HCs, MS patients demonstrated (1) reduced CBF‐ALFF coupling in the ALFF‐based analysis, and (2) increased CBF/ALFF ratios in the bilateral medial prefrontal cortex (mPFC). The regional mPFC finding, particularly on the left, remained robust across sensitivity analyses and was associated with worse cognitive performance and higher EDSS scores.

**Conclusion:**

These findings suggest altered NVC in MS, with regional prefrontal abnormalities representing the most robust and clinically relevant finding. Regional prefrontal NVC abnormalities may provide a potential imaging correlate of cognitive performance in MS.

## Graphical abstract

Multimodal MRI showed reduced global CBF‐ALFF coupling and increased medial prefrontal CBF/ALFF ratios in multiple sclerosis. The mPFC abnormality was associated with lower cognitive performance and greater disability.

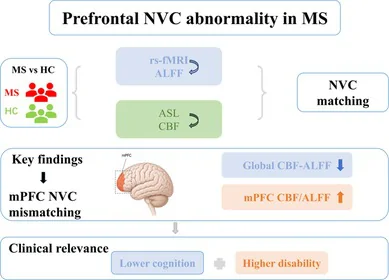

## 1 Introduction

Multiple sclerosis (MS), an immune‐mediated inflammatory demyelinating and neurodegenerative disorder of the central nervous system, represents the leading non‐traumatic cause of neurological disability in young adults [1]. Cognitive impairment is highly prevalent in MS, often emerging early in the disease course and significantly affecting patients' quality of life [2, 3, 4]. While extensive demyelination and gray matter disease are recognized contributors, the comprehensive etiology of cognitive impairment remains ambiguous [5, 6]. Critically, efficient cognitive function relies on the precise, moment‐to‐moment matching of CBF to neuronal metabolic demand—a process known as NVC [7]. Therefore, investigating whether NVC alterations are associated with cognitive performance may help clarify the neurovascular basis of cognitive changes in MS.

NVC is a key physiological process via which neuronal activity rapidly affects local CBF to power neural computation [8]. This critical process depends on the coordinated function of the neurovascular unit (NVU), which comprises vascular‐associated cells, including endothelial cells, astrocytes, and pericytes, as well as neurons and other glial cells that form the neurovascular microenvironment [9]. The NVU maintains cerebral homeostasis by dynamically regulating CBF, preserving blood–brain barrier (BBB) integrity, and modulating neuroinflammation [9, 10]. Critically, multiple components of the NVU are compromised in MS. The core inflammatory cascade in MS, driven by activated astrocytes and microglia, disrupts the BBB and facilitates the infiltration of immune cells [9, 11, 12, 13, 14]. Consequently, NVU disruption is a hallmark of MS pathology, providing a mechanistic rationale for examining whether NVC alterations are related to cognitive performance in MS.

Noninvasive assessment of NVC‐related physiology requires concurrent measures of spontaneous neuronal activity and cerebral perfusion. Resting‐state functional magnetic resonance imaging (fMRI) provides ALFF as a proxy for spontaneous neuronal activity [15, 16, 17, 18], whereas ASL allows quantitative assessment of CBF [19]. Because NVC reflects the matching between neuronal activity and vascular supply, the across‐voxel CBF‐ALFF correlation was used as an imaging‐derived index of global neurovascular coordination. The CBF/ALFF ratio was used to reflect regional blood supply relative to spontaneous neuronal activity. Reduced global coupling may indicate weaker large‐scale coordination, whereas deviations in the regional ratio in either direction may indicate neurovascular mismatch. These measures should be interpreted as indirect MRI‐based proxies of NVC rather than direct measures of NVU integrity. This approach has been applied in healthy populations and neurological disorders [20, 21, 22, 23].

Previous studies in MS have provided initial evidence for NVC alterations. Visual stimulation paradigms have linked NVC alterations to cognitive performance in MS [7, 24, 25, 26]. However, such task‐based paradigms can be challenging for patients with significant fatigue or cognitive deficits and may not fully capture the resting‐state neurovascular physiology. Furthermore, while unimodal studies have separately reported abnormalities in functional connectivity, perfusion, or neural activity in MS and linked them to clinical or cognitive measures [27, 28, 29], these metrics do not capture the coupling between vascular supply and neural activity. Thus, several issues remain unresolved: whether MS is associated with resting‐state NVC alterations at both global and regional levels, whether regional NVC mismatch occurs in cognition‐related hubs such as the mPFC, and whether the main NVC findings remain stable after accounting for structural and disease‐related factors.

Accordingly, this study examined multilevel NVC alterations in MS using concurrent resting‐state functional magnetic resonance imaging (rs‐fMRI) and ASL. We aimed to: (1) determine whether MS is associated with altered global CBF‐ALFF coupling; (2) identify regional NVC mismatch, particularly in cognition‐related hubs such as the mPFC, and examine its clinical relevance; and (3) test the robustness of the main findings after accounting for structural and disease‐related factors.

## 2 Materials and Methods

### 2.1 Participants

**FIGURE 1:**
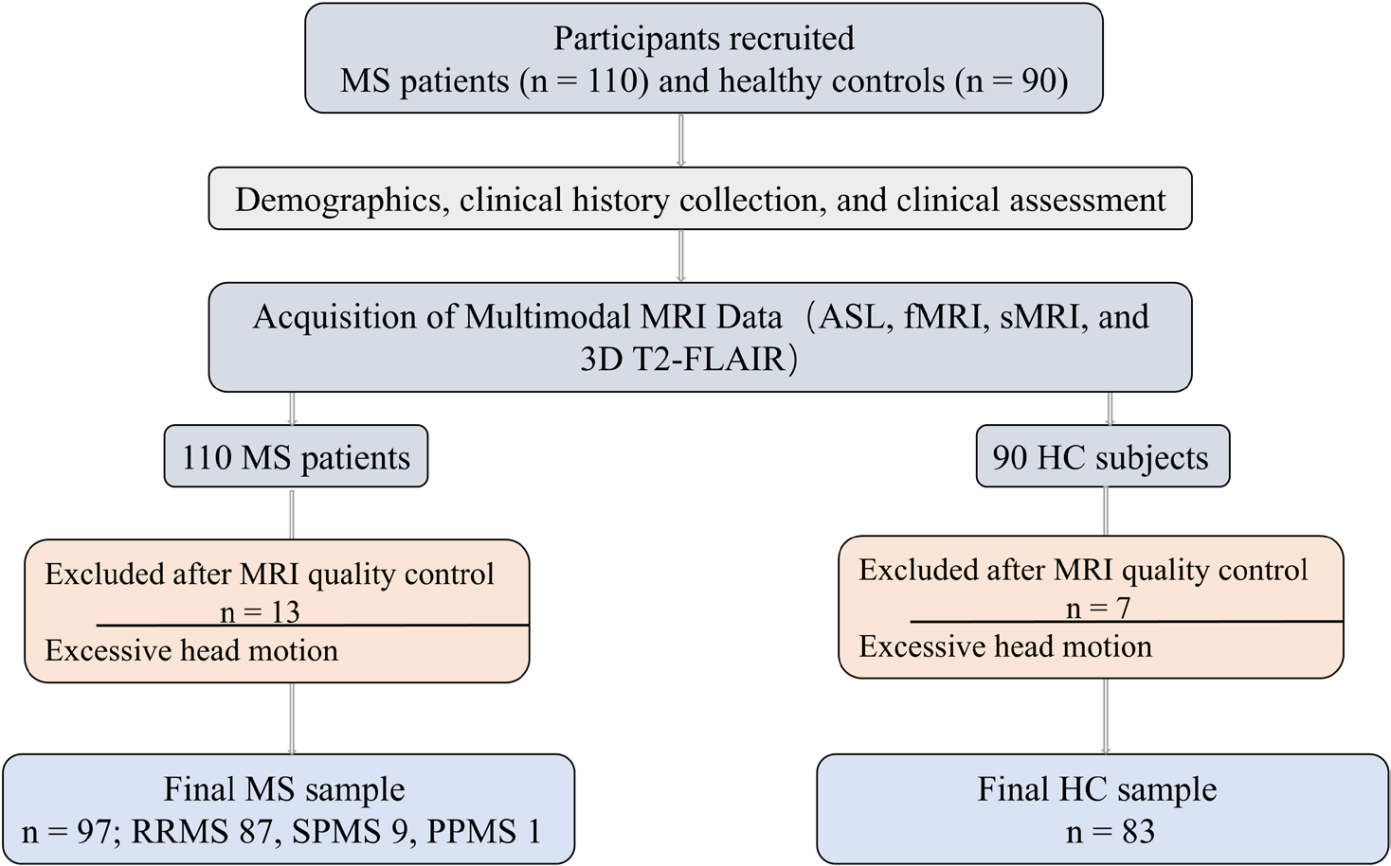
Flowchart of participant selection and study design. ASL, arterial spin labeling; fMRI, functional magnetic resonance imaging; HC, healthy control; MS, multiple sclerosis; sMRI, structural MRI.

This retrospective cross‐sectional study included 110 patients with clinically diagnosed MS who underwent multimodal brain MRI at the First Hospital of China Medical University between 2021 and 2025. Experienced neurologists diagnosed all patients, and eligibility for the present analysis was confirmed according to the 2024 McDonald criteria [30]. Eligible MS patients were right‐handed and had completed multimodal brain MRI. Additionally, 90 right‐handed healthy controls (HCs), matched for sex, age, and education, were included during the same period. Exclusion criteria for all participants were contraindications to MRI, a history of head trauma or other neuropsychiatric disorders, and poor image quality. Disease severity in patients with MS was assessed using the EDSS. After excluding 13 MS patients and 7 HCs because of image quality issues, 97 MS patients and 83 HCs were included in the final analysis. Among the 97 patients with MS, 87 had relapsing‐remitting MS (RRMS), 9 had secondary progressive MS (SPMS), and 1 had primary progressive MS (PPMS). None of the patients had received corticosteroids within 1 month before MRI scanning. The detailed flowchart is shown in Figure 1, and demographic and clinical information is summarized in Table 1.

**TABLE 1:** Demographic, clinical, and conventional neuroimaging data.

	MS (*n* = 97)	HC (*n* = 83)	Statistics	*p*
Demographic				
Age (years)	33 (26, 38.5)	35 (26, 42)	*U* = 4655.0	0.071
Sex (F/M)	68/29	68/15	*χ* ^2^ = 3.386	0.066
Education (years)	15 (11, 16)	15 (12, 16)	*U* = 4204.0	0.598
Radiological parameters				
T2 lesion volume (mm^3^)	18.67 (5.30, 27.12)	NA	NA	NA
Clinical characteristics				
Disease duration (months)	37 (13, 67)	NA	NA	NA
EDSS	2.5 (2, 3.5)	NA	NA	NA
MMSE	29 (28, 30)	30 (29, 30)	*U* = 5538.5	< 0.001
MoCA	26 (25, 27)	28 (27, 29)	*U* = 6487.0	< 0.001
SDMT	51 (48, 55) (89/97)^a^	64 (58, 68) (74/83)^a^	*U* = 5433.5	< 0.001
DMT‐treated patients, *n* (%)	76 (78.35%)	NA	NA	NA
MS phenotype, *n* (%)				
RRMS	87 (89.69%)	NA	NA	NA
SPMS	9 (9.28%)	NA	NA	NA
PPMS	1 (1.03%)	NA	NA	NA

*Note:* Continuous data are presented as median (interquartile range).

Abbreviations: DMT, disease‐modifying therapy; EDSS, expanded disability status scale; HC, healthy control; MMSE, mini‐mental state examination; MoCA, montreal cognitive assessment; MS, multiple sclerosis; NA, not applicable; PPMS, primary progressive MS; RRMS, relapsing‐remitting MS; SDMT, symbol digit modalities test; SPMS, secondary progressive MS.

^a^ Number of participants with available scores/total number of participants.

### 2.2 Neuropsychological Scale Assessments

All participants completed neuropsychological assessments before MRI scanning. The Montreal Cognitive Assessment (MoCA) and Mini‐Mental State Examination (MMSE) were used as screening instruments to assess cognitive function. The Symbol Digit Modalities Test (SDMT) was administered to assess information‐processing speed and working memory. SDMT data were unavailable for some participants; detailed results are presented in Table 1.

### 2.3 MRI Acquisition

All participants underwent sequential MRI acquisition on a 3.0 T scanner (General Electric, SIGNA Pioneer, Milwaukee, WI, USA) equipped with a 32‐channel head coil. The imaging protocol included: (1) resting‐state fMRI (rs‐fMRI) for BOLD images: repetition time (TR) = 2000 ms; echo time (TE) = 30 ms; flip angle = 90°; field of view (FOV) = 240 × 216 mm^2^; matrix = 64 × 64; thickness = 3.5 mm; 34 interleaved transverse slices; and 210 volumes; (2) three‐dimensional pseudo‐continuous ASL (3D‐pcASL): TR = 4861 ms; TE = 10.9 ms; flip angle = 111°; FOV = 240 × 240 mm^2^; matrix = 128 × 128; thickness = 4 mm; number of slices = 36; post label delay = 2025 ms; (3) Three‐dimensional (3D) high‐resolution T1‐weighted sequence: TR = 7.9 ms; TE = 2.9 ms; inversion time (TI) = 400 ms; flip angle = 12°; FOV = 240 × 240 mm^2^; matrix = 512 × 512; thickness = 1 mm; number of slices = 344; (4) 3D T2‐weighted fluid‐attenuated inversion recovery (FLAIR) imaging: TR = 6800 ms; TE = 11.7 ms; FOV = 240 × 216 mm^2^; slice thickness = 2.0 mm.

### 2.4 Multimodal Data Analysis

The data processing workflow comprised three main steps: rs‐fMRI preprocessing and metric calculation, CBF calculation, and lesion segmentation. Data processing was performed using the DPARSF toolbox (Data Processing Assistant for Resting‐State fMRI, version 5.4, http://www.restfmri.net/forum/DPARSF), SPM12 (http://www.fil.ion.ucl.ac.uk/spm/software/spm12) [31], and code written in MATLAB 2022b.

#### 2.4.1 Rs‐fMRI Imaging Preprocessing and Metrics Calculation

The preprocessing pipeline included the following steps: (1) Original DICOM data were converted into 3D NIfTI format. (2) The first 10 volumes were discarded to ensure signal stability. (3) The acquisition time of all voxels was aligned to the middle slice as the reference time point. (4) Subjects with excessive head motion (translation > 3 mm or rotation > 3°) were excluded. (5) The images were then normalized to the MNI template (resampling voxel size = 3 × 3 × 3 mm^3^) using T1 image unified segmentation. Subjects with poor registration quality were excluded. (6) Linear trends caused by scanner drift and participant adaptation were removed. (7) Nuisance signals, including 24 Friston head motion parameters, white matter, whole brain signals, and cerebrospinal fluid (CSF) signals, were regressed out. (8) Functional images were band‐pass filtered between 0.01 and 0.1 Hz.

##### 2.4.1.1 ALFF and fALFF Calculation

The amplitude of low‐frequency fluctuation (ALFF) and fractional ALFF (fALFF) were computed using the DPARSF toolbox (version 5.4). The preprocessed time series was transformed into the frequency domain via fast Fourier transform (FFT). The ALFF value was then defined as the average square root of the power spectrum within the 0.01–0.1 Hz frequency range after FFT application. The fALFF value was defined as the ratio of the power spectrum in the given frequency range to the entire frequency band. For standardization purposes, the ALFF/fALFF value for each voxel underwent Z‐transformation by subtracting the global mean value and then dividing by the standard deviation within the gray matter mask, and then spatially smoothed with a Gaussian kernel of 8 mm full‐width at half maximum (FWHM).

##### 2.4.1.2 ReHo Calculation

ReHo was calculated by computing Kendall's coefficient of concordance between the time series of each voxel and those of its 26 nearest neighboring voxels. Similarly, for standardization, the ReHo value for each voxel was Z‐transformed within the gray matter mask. Next, the zReHo maps were spatially smoothed with a Gaussian kernel of 8 mm FWHM.

#### 2.4.2 CBF Calculation and Analysis

CBF maps were processed as follows: (1) The 3D pcASL sequence automatically reconstructed CBF maps; each CBF map underwent visual inspection; subjects with poor image quality were excluded. (2) Raw DICOM files were converted to 3D NIfTI format using dcm2nii. (3) CBF images were normalized using SPM12 in MATLAB R2022b. (4) Normalized CBF maps were visually inspected to ensure proper alignment; subjects with misregistration were excluded. (5) Using the DPABI toolbox, the CBF maps were standardized by z‐transformation within the gray matter mask to reduce inter‐individual variability. (6) Normalized CBF maps were smoothed with an 8‐mm FWHM Gaussian kernel to improve signal‐to‐noise ratio (SNR) and ensure a Gaussian distribution for statistical analysis.

#### 2.4.3 Lesion Segmentation

T2 lesion volume (LV) for each participant was determined as follows. All 3D T2‐FLAIR images were spatially normalized to the MNI152 standard space using the Advanced Normalization Tools package (https://github.com/ANTsX). MS lesions were then segmented from the normalized images using the MoOME model [32], and total LV was calculated for each participant. To describe lesion distribution, lesion count and lesion volume were summarized across predefined anatomical locations, including subcortical, periventricular, infratentorial, and deep white matter regions.

### 2.5 Analysis of Whole Gray Matter Global CBF‐ALFF Coupling

For each participant, both CBF and ALFF maps were further normalized using a z‐transformation within the gray matter mask, enabling averaging and comparison across subjects. To assess the global CBF‐ALFF coupling across the whole gray matter, across‐voxel correlation analyses were conducted for each participant [17]. Group differences in CBF‐ALFF correlation coefficients were then compared using analysis of covariance (ANCOVA), with age, sex, and education included as covariates.

### 2.6 Analysis of CBF/ALFF Ratio Coupling

The ratio CBF/ALFF was computed for each voxel by dividing the CBF by the ALFF. To increase normality, the CBF/ALFF ratio of each voxel for each participant was transformed into a z‐score. ANCOVA was performed to detect differences in CBF/ALFF between the MS and HC groups, with age, sex, and education as covariates. Statistical significance was determined using family‐wise error correction at the cluster level (*p*_FWE_ < 0.05) with a voxel threshold of *p* < 0.001. The average CBF/ALFF ratio for each significant cluster was extracted for correlation analysis. Both the across‐voxel CBF‐ALFF correlation and the regional CBF/ALFF ratio were interpreted as indirect MRI‐based proxies of NVC, rather than direct measures of NVU integrity.

### 2.7 Voxel‐Wise CBF and ALFF Analyses

To further analyze the reasons for the difference in CBF/ALFF ratio between the MS and HC groups, voxel‐wise comparisons were performed to identify CBF and ALFF differences between the two groups, with age, sex, and education included as covariates. Voxel‐wise CBF and ALFF group differences were assessed using voxel‐wise FWE correction at *p* < 0.05. For the reduced‐ALFF contrast, results are reported at both the voxel‐wise FWE‐corrected threshold (*p* < 0.05) and an exploratory voxel‐wise FDR‐corrected threshold (*q* < 0.05). The FWE‐corrected results are provided in the Supporting Information, while the FDR‐corrected findings are presented in Table 2 and Figure 3 and are interpreted as exploratory.

**TABLE 2:** Principal brain regions showing significant intergroup differences in CBF, ALFF, and CBF/ALFF ratio.

Brain regions	Brodmann area	Peak MNI coordinates (*x*, *y*, *z*)	Peak *T* value	Cluster size (voxels)
*CBF*				
MS > HC				
Right lingual gyrus		18, –75, −3	5.5848	28
Left insula		−30, 12, –6	6.0751	133
Putamen				
Lentiform nucleus				
Right putamen		30, –12, 3	7.0736	380
Lentiform nucleus				
Insula				
Right medial prefrontal cortex	10	21, 57, 3	6.2382	158
Left putamen		−30, −12, 3	5.3287	23
Lentiform nucleus				
Right thalamus		18, –24, 6	5.2201	6
MS < HC				
Left inferior frontal gyrus	44/45	−57, 21, 15	−5.5907	37
Left precuneus	7	−6, −66, 45	−5.1773	24
*ALFF*				
MS > HC				
Right fusiform		42, –21, −24	5.0975	7
Left fusiform	36	−24, −39, −15	6.7808	34
Parahippocampal gyrus				
Right fusiform		24, –36, −18	5.6128	19
Parahippocampal gyrus				
Left fusiform		−36, −60, −3	5.0608	17
Right middle temporal gyrus		33, –30, 0	6.6062	111
Hippocampus				
Left middle temporal gyrus		−30, −36, 6	5.8299	51
Right calcarine		27, –51, 9	5.6038	24
MS < HC^a^				
Bilateral medial prefrontal cortex	10	3, 63, 18	−4.7703	225
Bilateral precuneus	7	−6, −84, 45	−5.1951	630
Superior parietal lobule				
*CBF/ALFF*				
MS > HC				
Left medial prefrontal cortex	10	−15, 54, 12	4.2833	215
Right medial prefrontal cortex	10	21, 51, 6	4.1677	122

^a^ Reduced ALFF was identified at an exploratory voxel‐wise FDR‐corrected threshold (*q* < 0.05). Results obtained at the stricter voxel‐wise FWE‐corrected threshold are presented in Figure S4 and Table S3.

**FIGURE 2:**
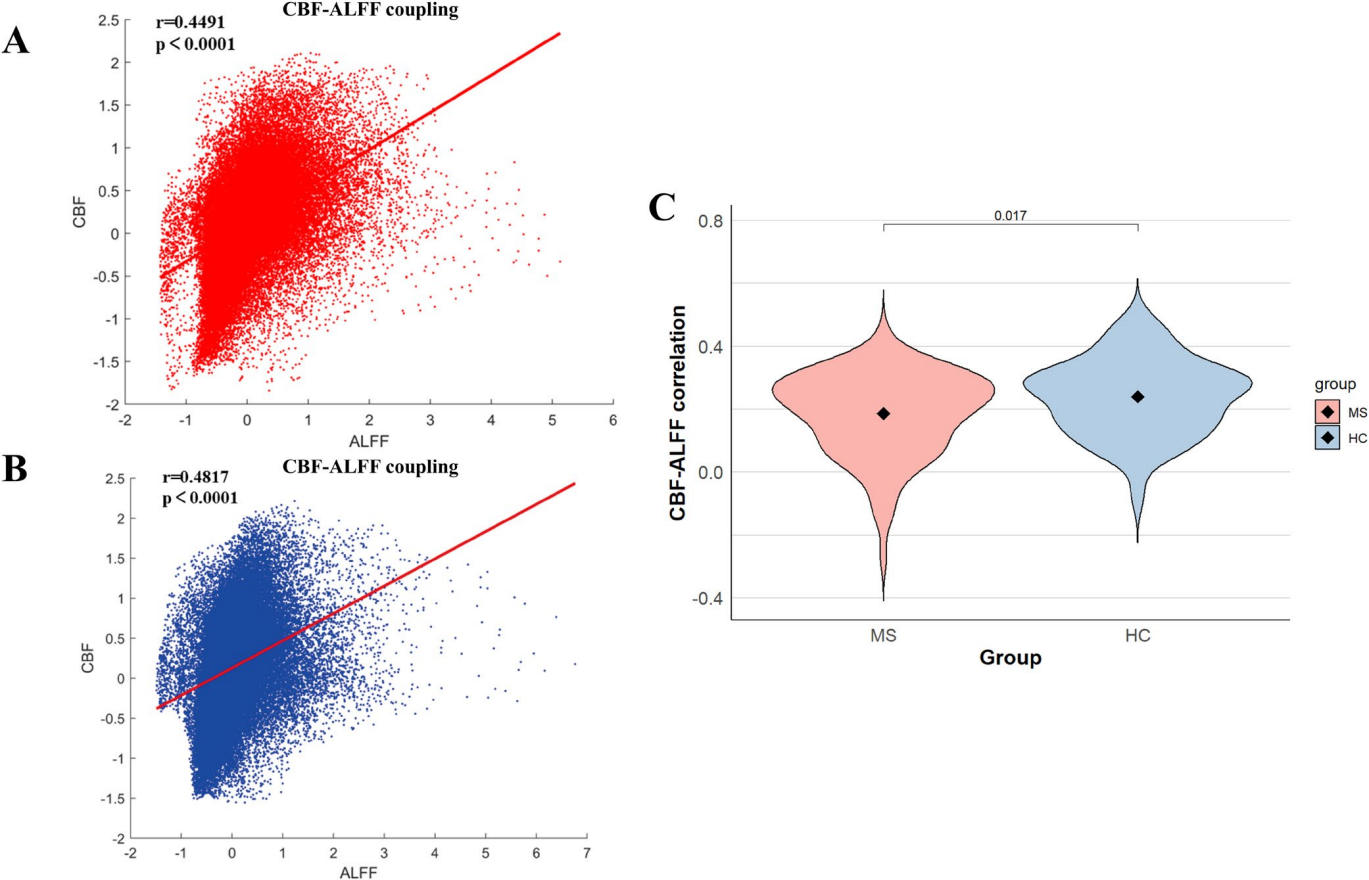
Reduced global CBF‐ALFF coupling in multiple sclerosis. (A, B) Examples of the spatial correlation across voxels between CBF and ALFF in a patient (red) and a control subject (blue). (C) At the group level, MS patients showed significantly reduced CBF‐ALFF coupling compared to HCs (*p* = 0.017). Abbreviations: ALFF, amplitude of low‐frequency fluctuations; CBF, cerebral blood flow; HC, healthy control; MS, multiple sclerosis.

### 2.8 Correlation Analyses

The average CBF/ALFF ratio for each significant cluster was extracted from the MS and HC groups using voxel‐wise comparisons. Spearman's correlation coefficients were employed to assess the relationship between CBF/ALFF ratios in these clusters and clinical scores in MS patients.

### 2.9 Sensitivity Analyses

To evaluate the robustness of the primary findings, several sensitivity analyses were performed.

#### 2.9.1 Robustness to Neuronal Activity Metric

We recomputed global and regional NVC metrics using fALFF and ReHo as alternative indicators of neuronal activity.

#### 2.9.2 Structural and Disease‐Related Factors

Particular attention was given to the potential influence of LV. Within the MS group, LV was incorporated into regression models to examine its association with regional NVC and to determine whether the relationship between NVC and cognitive performance remained after adjustment for LV. The main analyses were also repeated with mean GMV as an additional covariate, and supplementary subgroup analyses were conducted according to disease‐modifying therapy (DMT) status.

#### 2.9.3 Sample Stability Analyses

To reduce potential bias from disease severity, extreme values, and subtype composition, the analyses were repeated after excluding patients with EDSS ≥ 6 and participants with extreme CBF/ALFF values (below the 5th or above the 95th percentiles). Because the cohort was predominantly composed of RRMS patients, the principal analyses were also repeated in the RRMS subgroup.

#### 2.9.4 Exploratory MoCA‐Based Subgroup Analysis

As an exploratory supplementary analysis, MS patients were divided into MoCA < 26 and MoCA ≥ 26 subgroups [33]. Between‐group differences in demographic and clinical variables were assessed using Mann–Whitney *U* tests or χ^2^ tests, as appropriate. Bilateral mPFC CBF/ALFF ratios were compared using ANCOVA, with age, sex, and education as covariates.

## 3 Results

### 3.1 Demographic and Clinical Characteristics

A total of 97 MS patients (68 females, 29 males) and 83 HCs (68 females, 15 males) were included in this study. The demographic and clinical characteristics are summarized in Table 1. No significant differences were observed between the two groups in sex (χ^2^ = 3.386, *p* = 0.066), age (*U* = 4655.0, *p* = 0.071), or education (*U* = 4204.0, *p* = 0.598). Compared with HCs, MS patients had significantly lower MoCA, MMSE, and SDMT scores (all *p* < 0.001). Within the MS cohort, the median disease duration was 37 months, and the median EDSS score was 2.5. The cohort was predominantly RRMS. T2 lesion volume and other clinical characteristics are also summarized in Table 1.

### 3.2 Global CBF‐ALFF Coupling Changes in Patients With MS

The global CBF‐ALFF coupling differed between the two groups. MS patients showed significantly reduced global CBF‐ALFF coupling compared with HCs (0.186 ± 0.013 vs. 0.239 ± 0.013, *p* = 0.017; Figure 2).

**FIGURE 3:**
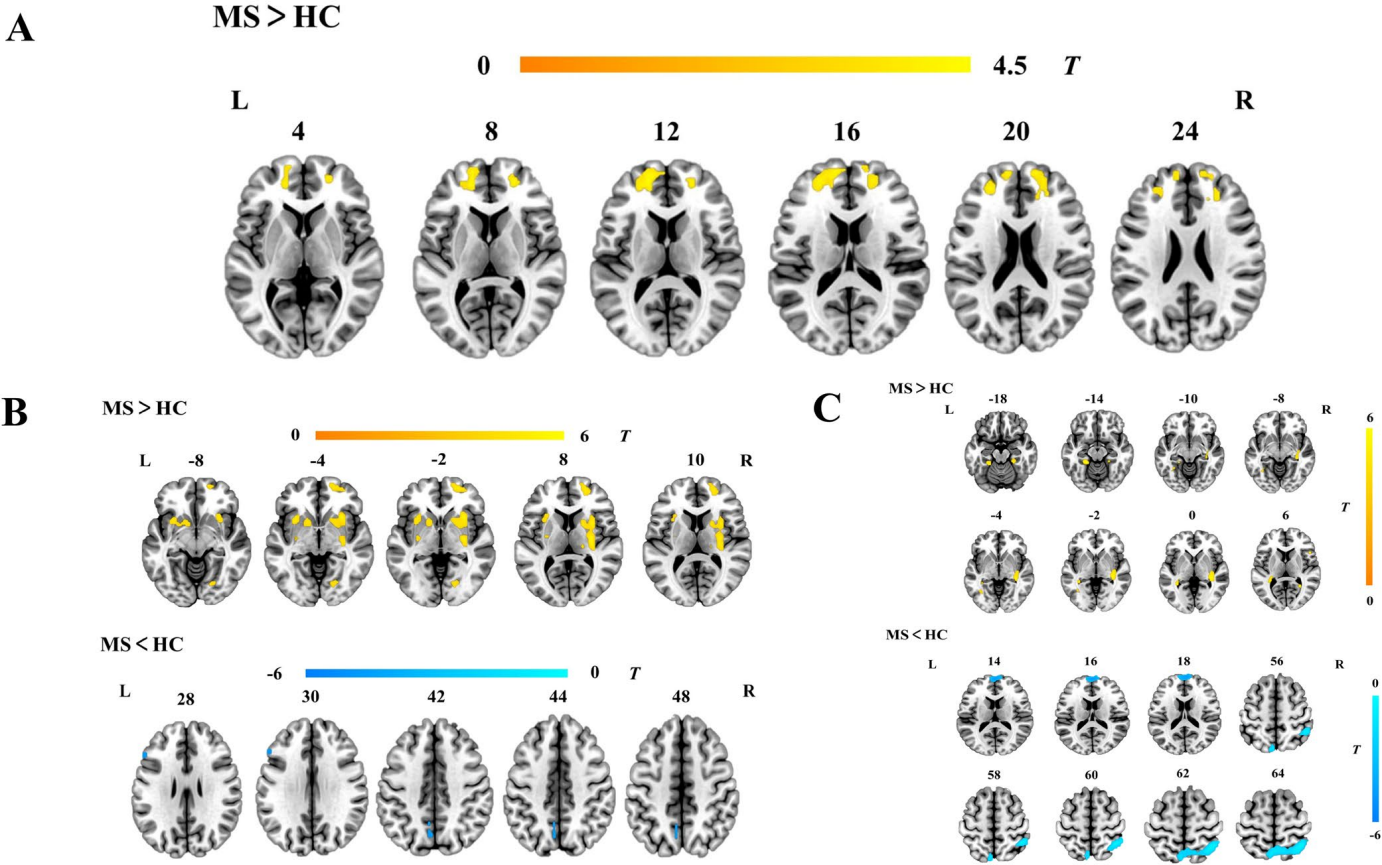
Regional alterations in neurovascular coupling and its hemodynamic and neuronal components in multiple sclerosis. Axial brain maps illustrate voxel‐wise group differences between MS patients and HCs. Warm colors indicate higher values in MS, and cool colors indicate lower values in MS. (A) CBF/ALFF ratio: MS patients exhibited increased CBF/ALFF ratios in the bilateral mPFC, indicating regional neurovascular mismatch (voxel‐level *p* < 0.001, uncorrected; cluster‐level *p* < 0.05, FWE‐corrected). (B) CBF: MS patients exhibited increased CBF in the bilateral basal ganglia and right mPFC, but decreased CBF in the left inferior frontal gyrus and precuneus (voxel‐wise FWE‐corrected *p* < 0.05). (C) ALFF: MS patients exhibited increased ALFF in the bilateral middle temporal, fusiform, and parahippocampal gyri (voxel‐wise FWE‐corrected *p* < 0.05). Reduced ALFF in the bilateral mPFC and precuneus was identified at an exploratory voxel‐wise FDR‐corrected threshold (*q* < 0.05). Results obtained at the stricter voxel‐wise FWE‐corrected threshold are presented in Figure S4 and Table S3. ALFF, amplitude of low‐frequency fluctuations; CBF, cerebral blood flow; FDR, false discovery rate; FWE, family‐wise error; HC, healthy control; mPFC, medial prefrontal cortex; MS, multiple sclerosis.

### 3.3 Regional CBF/ALFF Ratio Changes in Patients With MS

Compared with HCs, MS patients showed increased CBF/ALFF ratios in the bilateral mPFC (voxel‐level *p* < 0.001, uncorrected; cluster‐level *p* < 0.05, FWE‐corrected; Table 2 and Figure 3).

### 3.4 CBF and ALFF Changes in Patients With MS

Compared with HCs, MS patients showed higher CBF in the bilateral basal ganglia and right mPFC, and lower CBF in the left inferior frontal gyrus and precuneus (voxel‐wise FWE‐corrected *p* < 0.05; Table 2 and Figure 3).

For ALFF, MS patients showed higher values in the bilateral fusiform, parahippocampal, and middle temporal gyri at a voxel‐wise FWE‐corrected threshold (*p* < 0.05). At the exploratory voxel‐wise FDR‐corrected threshold, reduced ALFF was observed in the bilateral mPFC and precuneus (*q* < 0.05; Table 2 and Figure 3). Results obtained at the stricter voxel‐wise FWE‐corrected threshold are presented in Figure S4 and Table S3.

### 3.5 Associations With Clinical and Neuropsychological Measures

**FIGURE 4:**
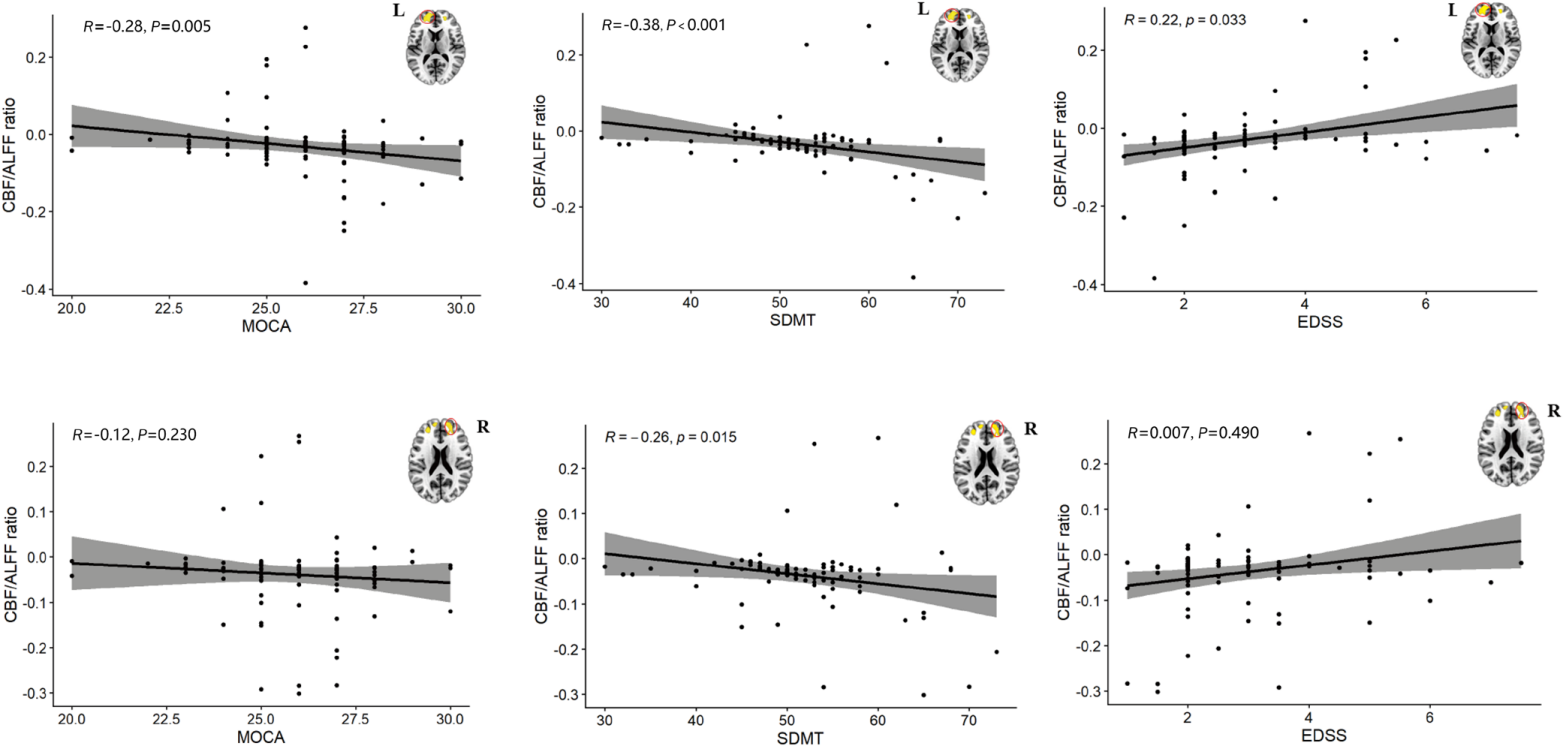
Correlations between regional NVC and EDSS scores and cognitive performance. The increased CBF/ALFF ratio in the left mPFC was negatively correlated with MoCA and SDMT and positively correlated with EDSS scores. The increased CBF/ALFF ratio in the right mPFC was negatively correlated with SDMT. ALFF, amplitude of low‐frequency fluctuations; CBF, cerebral blood flow; EDSS, expanded disability status scale; MoCA, Montreal Cognitive Assessment; mPFC, medial prefrontal cortex; SDMT, symbol digit modalities test.

Spearman correlations between the CBF/ALFF ratio of bilateral mPFC and EDSS scores and neuropsychological scores in MS are shown in Figure 4. In MS patients, the CBF/ALFF ratio in the left mPFC showed a negative correlation with MoCA (*r* = −0.28, *p* = 0.005) and SDMT scores (*r* = −0.38, *p* < 0.001), and a positive correlation with EDSS (*r* = 0.22, *p* = 0.033). Similarly, the right mPFC demonstrated a negative correlation with SDMT scores (*r* = −0.26, *p* = 0.015).

### 3.6 Sensitivity Analyses

#### 3.6.1 Robustness to Neuronal Activity Metric

When fALFF and ReHo were used as alternative neuronal activity metrics, the reduced global coupling observed in the ALFF‐based analysis was not reproduced (*p* = 0.078 and *p* = 0.087, respectively). In contrast, regional analyses continued to show increased CBF/fALFF and CBF/ReHo ratios specifically in the left mPFC in MS patients relative to HCs (both *p* < 0.005, uncorrected; Figure S1), further supporting the left mPFC abnormality as the most consistent regional finding across metrics.

#### 3.6.2 Structural and Disease‐Related Factors

Within the MS group, T2 lesion volume was not significantly associated with the CBF/ALFF ratio in either the left mPFC (model F(4,92) = 0.932, *p* = 0.449; LV: standardized *β* = 0.010, *p* = 0.924) or the right mPFC (model F(4,92) = 0.779, *p* = 0.542; LV: standardized *β* = −0.064, *p* = 0.538). Moreover, after adjustment for lesion volume, the association between bilateral mPFC CBF/ALFF ratio and SDMT performance remained significant, whereas lesion volume was not (Table 3). Location‐specific lesion burden is summarized in Table S1. Periventricular lesions accounted for the largest proportion of summed lesion volume, whereas deep white matter and subcortical lesions accounted for most lesion counts.

**TABLE 3:** Multiple linear regression analyses of T2 lesion volume, regional NVC, and SDMT performance in MS patients.

(A) Multiple linear regression analysis of the association between T2 lesion volume and regional NVC in MS patients							
Dependent variable	predictor	Unstandardized *β*	Standardized *β*	p	Model *F*(df)	Model *P*	*R* ^2^
left mPFC CBF/ALFF ratio	T2 lesion volume	4.59E‐05	9.91E‐03	0.924	0.932 (4,92)	0.449	0.039
right mPFC CBF/ALFF ratio	T2 lesion volume	−2.98E‐04	−0.064	0.538	0.779 (4,92)	0.542	0.033

(B) Multiple linear regression models for SDMT performance in MS patients					
Models and predictors	Unstandardized *β*	Standardized *β*	*p*	VIF	*R* ^2^_adj_
Model 1: Left mPFC					
Constant	45.235		**< 0.001**		0.117
Left mPFC CBF/ALFF ratio	−33.909	−0.316	**0.003**	1.049	
T2 lesion volume	−0.034	−0.077	0.454	1.038	
Age	−0.075	−0.102	0.321	1.035	
Sex	2.496	0.146	0.160	1.056	
Education	0.498	0.197	0.065	1.110	
Model 2: Right mPFC					
Constant	46.192		**< 0.001**		0.115
Right mPFC CBF/ALFF ratio	−34.238	−0.309	**0.003**	1.024	
T2 lesion volume	−0.037	−0.084	0.412	1.037	
Age	−0.082	−0.112	0.276	1.028	
Sex	2.457	0.144	0.167	1.055	
Education	0.434	0.172	0.104	1.096	

*Note:* In panel A, models were adjusted for age, sex, and years of education. In panel B, SDMT score was entered as the dependent variable; predictors included the CBF/ALFF ratio in the left or right mPFC, T2 lesion volume, age, sex, and years of education. Bold values indicate statistical significance at *p* < 0.05.

Abbreviations: ALFF, amplitude of low‐frequency fluctuations; CBF, cerebral blood flow; mPFC, medial prefrontal cortex; NVC, neurovascular coupling; SDMT, symbol digit modalities test.

Similarly, the main findings were preserved after controlling for GMV and across DMT subgroups. After further adjustment for mean GMV, the MS group continued to show significantly reduced global CBF‐ALFF coupling relative to HCs (*p* = 0.033), and the increased CBF/ALFF ratio in the left mPFC remained significant (voxel‐level *p* < 0.001, uncorrected; cluster‐level *p* < 0.05, FWE‐corrected) (Figure S2). In addition, both DMT‐treated and untreated MS patients showed significantly higher bilateral mPFC ratios than HCs, whereas no difference was observed between the two MS subgroups (left: F(2,175) = 10.069, *p* < 0.001; right: F(2,175) = 8.047, *p* < 0.001) (Figure S2).

#### 3.6.3 Sample Stability Analyses

To assess whether disease severity or extreme values confounded our findings, we performed two separate sensitivity analyses. Excluding five patients with severe disability (EDSS ≥ 6) did not alter the reduced global coupling (*p* = 0.030) or the spatial pattern of regional alterations (Figure S3). Likewise, removing seven MS patients and six HCs with extreme CBF/ALFF values (below the 5th or above the 95th percentiles) left both the global coupling reduction (*p* = 0.009) and the increased left mPFC ratio unchanged (voxel‐level *p* < 0.001, uncorrected; cluster‐level *p* < 0.05, FWE‐corrected) (Figure S3).

**FIGURE 5:**
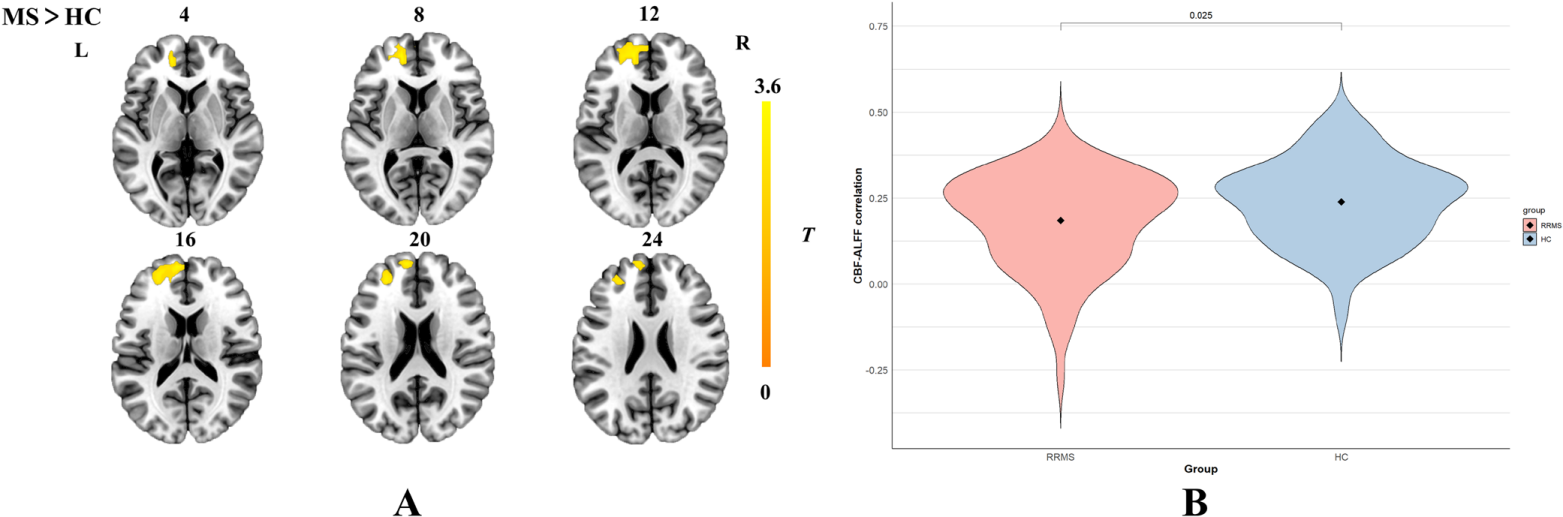
RRMS subgroup analysis of global and regional neurovascular coupling. (A) Global CBF‐ALFF coupling was significantly reduced in patients with relapsing‐remitting multiple sclerosis (RRMS) compared with healthy controls (HCs) (0.184 ± 0.015 vs. 0.239 ± 0.013, F = 6.463, *p* = 0.025), after adjustment for age, sex, and years of education. (B) Voxel‐wise analysis showed a significantly increased CBF/ALFF ratio in the left mPFC in RRMS relative to HCs (voxel‐level *p* < 0.001, uncorrected; cluster‐level *p* < 0.05, FWE‐corrected). ALFF, amplitude of low‐frequency fluctuations; CBF, cerebral blood flow; HC, healthy control; mPFC, medial prefrontal cortex; RRMS, relapsing‐remitting multiple sclerosis.

Because the cohort was predominantly composed of RRMS patients, the principal analyses were further repeated in the RRMS subgroup. The main findings were preserved, including reduced global CBF‐ALFF coupling in RRMS relative to HCs (0.184 ± 0.015 vs. 0.239 ± 0.013, F = 6.463, *p* = 0.025) and an increased CBF/ALFF ratio in the left mPFC (voxel‐level *p* < 0.001, uncorrected; cluster‐level *p* < 0.05, FWE‐corrected) (Figure 5).

#### 3.6.4 Exploratory MoCA‐Based Subgroup Analysis

In the exploratory MoCA‐based subgroup analysis, 38 patients were classified into the MoCA< 26 subgroup and 59 into the MoCA≥ 26 subgroup. After adjustment for age, sex, and education, no significant subgroup difference was observed in the left or right mPFC CBF/ALFF ratio (left: F(1,92) = 3.803, *p* = 0.054; right: F(1,92) = 0.311, *p* = 0.578; Table S2).

## 4 Discussion

This study employed multimodal ASL and BOLD imaging to examine alterations in multilevel neurovascular coupling in MS. We observed reduced global CBF‐ALFF coupling and, at the regional level, increased CBF/ALFF ratios in the bilateral mPFC. Importantly, the regional mPFC mismatch was associated with worse cognitive performance and higher EDSS scores, supporting its clinical relevance. Overall, the bilateral mPFC mismatch was the most consistent and clinically relevant finding.

Beyond the ALFF‐based global finding, the most prominent regional result was the abnormal CBF/ALFF ratio in the bilateral mPFC, a key hub of the default mode network and a region involved in higher‐order cognition [34]. The CBF/ALFF ratio reflects blood supply relative to spontaneous neural activity. It should not be interpreted as a unidirectional marker of NVC integrity. Instead, deviations in either direction may indicate neurovascular mismatch [21, 22, 23]. An elevated value may reflect excess blood flow relative to neuronal activity, suggesting a maladaptive mismatch, whereas a reduced value may suggest insufficient perfusion. In the present study, increased CBF/ALFF ratios were observed in both the left and right mPFC. At the exploratory FDR‐corrected threshold, reduced ALFF was observed in the bilateral mPFC, with a smaller medial prefrontal focus remaining significant after voxel‐wise FWE correction. These findings suggest that reduced spontaneous neuronal activity may contribute to the prefrontal mismatch, although the broader bilateral pattern should be interpreted cautiously. The right mPFC also showed elevated CBF, which may indicate an additional vascular component, such as altered perfusion regulation or inflammation‐related hyperperfusion [35, 36]. Thus, the regional findings suggest a possible neuronal contribution to the mPFC mismatch, whereas the right‐sided effect appeared to include a stronger perfusion contribution. This difference may also explain why the left mPFC finding was more stable across sensitivity analyses, while the right‐sided finding was more sensitive to perfusion‐related variability.

The clinical relevance of the mPFC finding is supported by its associations with both cognition and disability. This is particularly important because the mPFC is a key hub for higher‐order cognitive processing and large‐scale network integration [34, 37]. In our cohort, higher CBF/ALFF ratios in the left mPFC were associated with lower MoCA and SDMT scores and with higher EDSS scores, whereas the right mPFC was also negatively associated with SDMT performance. Because information processing speed is commonly affected in MS [5], and SDMT is a sensitive indicator of this domain [38], these results suggest that prefrontal NVC mismatch is not merely an imaging finding, but is related to worse cognitive performance and disease severity. An exploratory MoCA‐based subgroup analysis did not show significant adjusted differences in bilateral mPFC CBF/ALFF ratios; therefore, the main interpretation remains focused on continuous cognitive‐performance associations rather than categorical cognitive impairment. Together with the bilateral regional pattern described above, these findings support the mPFC mismatch as the most clinically informative result of the present study.

The reduced global CBF‐ALFF coupling observed in MS may reflect disturbed large‐scale neurovascular coordination. In the intact brain, the substantial across‐voxel correlation between CBF and ALFF is thought to reflect the close matching between vascular supply and spontaneous neuronal activity [17, 20]. Disruption of NVU integrity, including astrocytic dysfunction, demyelination‐related neuronal injury, and blood–brain barrier abnormalities, may therefore contribute to this global decoupling in MS [9, 11, 12, 13, 14, 39]. However, this finding should be interpreted with caution, because it was not consistently reproduced when fALFF or ReHo were used as alternative functional metrics. This metric dependence is biologically plausible, as ALFF reflects the amplitude of low‐frequency BOLD fluctuations [18], fALFF emphasizes normalized low‐frequency spectral contribution [40], and ReHo indexes local temporal synchrony rather than amplitude itself [41]. Accordingly, the ALFF‐based global result may indicate that the observed effect is more closely related to altered fluctuation amplitude than to normalized spectral composition or local synchrony. Even so, the regional mPFC abnormality remained the most consistent finding of the present study.

The stability of the mPFC finding across sensitivity analyses suggests that it is unlikely to be a secondary effect of other disease‐related factors. This was most clearly reflected in the lesion volume and subtype analyses, both of which address central concerns in MS research. The absence of a significant relationship between T2 lesion volume and mPFC CBF/ALFF ratio, together with the persistence of its association with cognitive performance after adjustment for lesion volume, argues against macroscopic lesion volume as the main explanation. However, total lesion volume does not capture lesion topography. Lesion location may partly contribute to clinical and cognitive heterogeneity in MS, but its explanatory value appears limited. Thus, location‐specific effects should be considered when interpreting regional NVC findings [42, 43]. Likewise, the preservation of the principal findings in the dominant RRMS subgroup makes it less likely that they were driven by mixed subtype composition. Although the sensitivity analyses were not fully uniform, they generally supported the regional mPFC result. In particular, the left‐sided abnormality remained evident across several complementary analyses and appeared to be the more stable component of the bilateral pattern.

Several limitations should be noted. First, the cross‐sectional design precludes causal inference. Longitudinal studies are needed to determine whether NVC abnormalities precede, accompany, or follow changes in cognitive performance. Second, as in previous multimodal MRI studies, the CBF/ALFF ratio and global CBF‐ALFF correlation indirectly reflect NVC rather than directly measuring NVU integrity [21]. The underlying biological mechanisms, therefore, remain to be clarified. Third, although the cognitive assessment covered global cognition and processing speed, a broader neuropsychological battery would better characterize domain‐specific cognitive performance. The small number of progressive MS patients also limits generalizability beyond the dominant RRMS subgroup. Although total T2 lesion volume was considered and location‐specific lesion burden was summarized descriptively, lesion location was not modeled in detail. Because detailed lesion‐topography analysis requires dedicated spatial methods, future lesion‐symptom mapping or tract‐based studies are needed to clarify its contribution to regional NVC‐related findings [44]. We also did not apply lesion filling; although this approach may reduce inter‐subject variability, it can introduce artifacts and inaccuracies in patients with higher lesion loads [45]. Finally, although a smaller medial prefrontal focus survived voxel‐wise FWE correction, the broader spatial distribution of ALFF reductions was identified at an exploratory FDR‐corrected threshold and requires confirmation in independent cohorts. Despite these limitations, the present findings suggest that regional mPFC NVC abnormality is a clinically relevant feature of MS.

## 5 Conclusion

Our findings suggest multilevel NVC alterations in MS, with regional prefrontal abnormalities representing the most robust and clinically relevant result. Although the cross‐sectional design does not permit causal inference, these abnormalities were associated with worse cognitive performance and disability. Future longitudinal studies are needed to clarify their temporal and prognostic relevance, and interventional studies targeting NVU integrity may help determine whether improving neurovascular function is associated with better cognitive outcomes in MS.

## Author Contributions

Ruisi Gong wrote the text, conducted preliminary analysis, and gathered data. Data analysis was overseen by Hao Zhang and Jinlin Jiao. Lei Liu, Xiaohan Xu, Huiwen Song, Yuexin Zhou, and Yuanjie Duan were responsible for the investigation and data collection. Lingling Cui, Jibin Cao, and Hefei Fu revised the manuscript, in addition to conceptualizing the study design. All authors read and approved the final manuscript.

## Funding

This work was supported by the Science and Technology Project of Liaoning Province, China (No. 2023JH2/20200020 to Lingling Cui) and the National Innovation Center for Advanced Medical Devices (No. NMED2025CX‐01‐003 to Jibin Cao).

## Ethics Statement

This retrospective study was approved by the Medical Research Ethics Committee of the First Hospital of China Medical University (approval No. [2024]1126). All procedures involving human participants were performed in accordance with the Declaration of Helsinki and its later amendments. Written informed consent was obtained from all participants.

## Consent

The authors have nothing to report.

## Conflicts of Interest

Cui and Cao report receiving grant support from the Science and Technology Project of Liaoning Province and the National Innovation Center for Advanced Medical Devices for this study (see Funding Statement) and declare no financial conflicts of interest with any commercial entity. The other authors declare no potential conflicts of interest with respect to the research, authorship, and/or publication of this article.

## Supporting information


**Figure S1:** Sensitivity analyses using alternative neuronal activity metrics.
**Figure S2:** Effects of GMV and DMT on mPFC CBF/ALFF ratios.
**Figure S3:** Sample stability analyses after excluding severe cases and extreme values.
**Figure S4:** ALFF reductions in patients with multiple sclerosis.
**Table S1:** Location‐specific lesion burden in patients with MS.
**Table S2:** Exploratory MoCA‐based subgroup analysis in patients with MS.
**Table S3:** Brain regions showing lower ALFF in patients with MS than in HCs.

## Data Availability

Code used for image processing and statistical analyses is available from the corresponding author upon reasonable request.
