# Supplementary material for: Prefrontal Neurovascular Coupling Abnormalities Associated With Cognitive Performance in Multiple Sclerosis
Source: CNS Neurosci Ther. 2026 Aug 31;32(9):e71124. doi: 10.1002/cns.71124 (PMC13530862; doi:10.1002/cns.71124)
Supplement: Supplementary file 1 [file CNS-32-e71124-s001.doc]

**Supplement Materials**

**Supplementary Figure S1. Sensitivity analyses using alternative neuronal activity metrics**

(A) At the group level, no significant differences were found in global CBF-ReHo coupling between MS patients and HCs (*p* = 0.087).

(B) At the group level, no significant differences were found in global CBF-fALFF coupling between MS patients and HCs (*p* = 0.078).

(C) Group differences in CBF/ReHo ratio between MS and HCs.

The spatial patterns of increased regions largely replicate the main findings derived from the CBF/ALFF ratio, supporting the robustness of the regional findings.

(D) Group differences in CBF/fALFF ratio between MS and HCs.

The spatial patterns of increased regions largely replicate the main findings derived from the CBF/ALFF ratio, supporting the robustness of the regional findings.

Abbreviations: ALFF, amplitude of low-frequency fluctuations; CBF, cerebral blood flow; fALFF, fractional ALFF; ReHo, regional homogeneity; MS, multiple sclerosis; HC, healthy control.

**Supplementary Figure S2. Effects of GMV and DMT on mPFC CBF/ALFF ratios**

(A) The left mPFC CBF/ALFF ratio remained significantly increased after GMV adjustment (voxel-level *p* < 0.001, uncorrected; cluster-level *p* < 0.05, FWE-corrected).

(B, C) Both DMT-treated and untreated MS subgroups showed higher bilateral mPFC ratios than healthy controls, with no differences between the two MS subgroups (left: F (2,175) = 10.069, *p* < 0.001; right: F (2,175) = 8.047, *p* < 0.001). Bonferroni correction was applied to post hoc comparisons.

Abbreviations: ALFF, amplitude of low-frequency fluctuations; CBF, cerebral blood flow; GMV, gray Matter Volume; DMT, disease-modifying therapy; MS, multiple sclerosis; HC, healthy control; ns, not significant.

**Supplementary Figure S3. Sample stability analyses after excluding severe cases and extreme values**

(A, C) Excluding patients with EDSS ≥ 6 did not materially alter the reduced global CBF-ALFF coupling (*p* = 0.030) or the regional pattern of abnormalities.

(B, D) Excluding participants with extreme CBF/ALFF values also preserved the reduced global coupling (*p* = 0.009) and the increased left mPFC CBF/ALFF ratio (voxel-level *p* < 0.001, uncorrected; cluster-level *p* < 0.05, FWE-corrected).

Abbreviations: ALFF, amplitude of low-frequency fluctuations; CBF, cerebral blood flow; EDSS, Expanded Disability Status Scale; MS, multiple sclerosis; HC, healthy control.

**Supplementary Figure S4. ALFF reductions in patients with multiple sclerosis**

Axial brain maps show regions with lower ALFF in patients with MS than in HCs at voxel-wise FWE-corrected *p* < 0.05. Cool colors indicate lower ALFF in MS.

Abbreviations: ALFF, amplitude of low-frequency fluctuations; FWE, family-wise error; HC, healthy control; MS, multiple sclerosis.

**Supplementary Table S1**. Location-specific lesion burden in patients with MS

| **Lesion location** | **Patients with lesions, n (%)** | **Lesion count, median (IQR)** | **Total lesion count, n (%)** | **Lesion volume, mm³, median (IQR)** | **Summed lesion volume, mm³ (%)** |
| --- | --- | --- | --- | --- | --- |
| Subcortical | 97 (100.0) | 17.0 (7.0-29.0) | 2107 (39.7) | 1.44 (0.70-3.30) | 358.45 (19.5) |
| Periventricular | 97 (100.0) | 7.0 (5.0-9.0) | 666 (12.6) | 11.02 (2.54-20.99) | 1366.91 (74.3) |
| Infratentorial | 84 (86.6) | 2.0 (1.0-3.0) | 233 (4.4) | 0.11 (0.01-0.31) | 26.96 (1.5) |
| Deep white matter | 97 (100.0) | 20.0 (12.0-29.0) | 2298 (43.3) | 0.78 (0.37-1.15) | 88.28 (4.8) |

Note: Data are presented as median (interquartile range, IQR) unless otherwise indicated. Median and IQR were calculated across all 97 patients with MS. Percentages of total lesion count and total lesion volume were calculated relative to the summed lesion burden across the four predefined anatomical locations. Percentages may not sum to 100 because of rounding.

Abbreviations: MS, multiple sclerosis; IQR, interquartile range.

Supplementary Table S2. Exploratory MoCA-based subgroup analysis in patients with MS

| **Variable** | **MoCA < 26 (n = 38)** | **MoCA ≥ 26 (n = 59)** | **Statistic** | ***p* value** |
| --- | --- | --- | --- | --- |
| Age (years) | 33(28.75, 41.25) | 31(24, 35) | U = 853.0 | 0.047 |
| Sex (F/M) | 29/9 | 39/20 | χ^2^ = 1.151 | 0.283 |
| Education (years) | 15(12, 16) | 15(9, 16) | U = 1199.5 | 0.549 |
| T2 lesion volume (mm^3^) | 14.81(3.83, 25.94) | 16.32(5.47, 27.98) | U = 1221.5 | 0.458 |
| Disease duration (months) | 39(15, 74.25) | 37(13, 64) | U = 1058.5 | 0.644 |
| EDSS | 3(2, 5) | 2(2, 3) | U = 723.0 | 0.002 |
| Left mPFC CBF/ALFF ratio, unadjusted | -0.024(-0.036, -0.011) | -0.029(-0.057, -0.022) | U = 785.0 | 0.013 |
| Right mPFC CBF/ALFF ratio, unadjusted | -0.028(-0.052, -0.021) | -0.028(-0.052, -0.022) | U = 1005.0 | 0.391 |
| Left mPFC CBF/ALFF ratio, adjusted | -0.014 ± 0.014 | -0.048 ± 0.011 | F = 3.803 | 0.054 |
| Right mPFC CBF/ALFF ratio, adjusted | -0.039 ± 0.014 | -0.048 ± 0.011 | F = 0.311 | 0.578 |

Note: Data are presented as median (interquartile range, IQR), n, or adjusted mean ± standard error, as appropriate. Unadjusted comparisons were performed using Mann-Whitney U tests or χ^2^ tests. Adjusted comparisons of mPFC CBF/ALFF ratios were performed using ANCOVA with age, sex, and education as covariates.

Abbreviations: MS, multiple sclerosis; MoCA, Montreal Cognitive Assessment; EDSS, Expanded Disability Status Scale; mPFC, medial prefrontal cortex; CBF, cerebral blood flow; ALFF, amplitude of low-frequency fluctuations; ANCOVA, analysis of covariance; IQR, interquartile range.

**Supplementary Table S3. Brain regions showing lower ALFF in patients with MS than in HCs**

| **Brain regions** | **Brodmann area** | **Peak MNI coordinates (x, y, z)** | **Peak T value** | **Cluster size (voxels)** |
| --- | --- | --- | --- | --- |
| Left superior occipital gyrus  precuneus | 7 | −6 −84 45 | −5.1951 | 12 |
| Right superior parietal lobule | 7 | 36 −51 63 | −4.8710 | 7 |
| Bilateral medial prefrontal cortex | 10 | 3 63 18 | −4.7703 | 2 |
| Right superior parietal lobule |  | 45 −48 57 | −4.6709 | 1 |

Note: Negative T values indicate lower ALFF in MS than in HCs. Statistical significance was set at voxel-wise FWE-corrected *p* < 0.05.

Abbreviations: ALFF, amplitude of low-frequency fluctuations; FWE, family-wise error; HC, healthy control; MNI, Montreal Neurological Institute; MS, multiple sclerosis.
